# Dietary Fiber and WHO Food Categories Extension for the Food-Pics_Extended Database

**DOI:** 10.3389/fpsyg.2022.818471

**Published:** 2022-06-10

**Authors:** Evelyn Medawar, Ronja Thieleking, A. Veronica Witte

**Affiliations:** Max Planck Institute for Human Cognitive and Brain Sciences, Leipzig, Germany

**Keywords:** dietary fiber, food category, macronutrient, non-digestible carbohydrates, public health, food type

## Abstract

Well characterized databases used for experimental purposes with extensive meta-data are essential for conducting meaningful and comparable studies. The Food-pics_extended database (Blechert et al., 2019) is one example for a widely used food stimulus database (original publication Blechert et al., 2014: 285 citations, and 2019: 32 citations). Indeed, meta-data on low level and high level image characteristics is broad, yet fiber ratings are not included, limiting its use in diet-related studies. Therefore, we developed fiber ratings per item (*n* = 562 stimuli), based on mean values of four non-expert raters. Ratings show good reliability (ICC = 0.77) and meaningful ranges per food type (mean fiber per 100 g by food type min_beverages_ = 0.04 ± 0.04 g and max_*Ready–to–eat*_ _savories_ = 4.49 ± 1.58). The newly provided fiber ratings enrich the already valuable database and extend it by an important nutrient value for human and planetary health.

## Introduction

The rich Food-pics database extended database (Blechert et al., 2019) contains 568 well-characterized standardized food images that come along with extensive meta-data, i.e., besides ratings on general palatability, craving, arousal, and visual image information also nutritional information, such as calories, carbohydrates, protein and fat relative to 100 g servings and per depicted portion. However, information on dietary fiber content is not available, somewhat limiting the database’s use in nutrition and psychological research.

### The Importance of Dietary Fiber for Health

Dietary fiber is regarded essential to a healthy diet, and the World Health Organization (WHO) and European nutritional agencies recommend a daily intake of > 25 g. The average European citizen with a daily intake of ∼16–24 g does not reach these recommendations though (Stephen et al., 2017). Known health benefits of adequate fiber intake comprise improved colonic function, blood cholesterol and blood glucose, cardiovascular health, and benefits for conditions like type-2 diabetes, obesity and certain types of cancer (Stephen et al., 2017). A large meta-analysis across 185 prospective studies and 58 clinical trials concluded that food items rich in whole-grain or dietary fiber are complementary in mediating improvements in non-communicable diseases in a dose-response relationship (Reynolds et al., 2019).

### The Common “Fiber Knowledge Gap”

Commonly consumed food products with high fiber content include whole grain products, bread, potatoes, vegetables, legumes, and fruits, however nutrition facts on food packaging do not always report dietary fiber and its awareness in public nutrition literacy is often low. For instance in the EU, while labeling for the “Big 7” is mandatory (i.e., kJ/kcal, fat, saturated fatty acids, carbohydrates, sugar, protein, salt) labeling for additional nutrients (e.g., unsaturated fats, fiber, starch, vitamins, minerals) is voluntary only [Regulation (EU) No 1169/2011^1^ ]. In contrast, in the United States the FDA specifies to list dietary fiber and total sugars as nutritional subcategories (FDA, 2015^2^).

In sum, their broad health implications together with a relatively fragmentary availability of educative information urges the need to increase both awareness and tailored research on dietary fiber-related questions, for example in the context of food choices and eating behavior. Therefore, we aimed to extend the Food-pics_extended database with readily usable dietary fiber information.

## Method

### Food Category Extension for the Food-Pics_Extended Database

To provide this information, we first added new standardized food categories to the Food-pics_extended database according to the WHO and Food and Agriculture Organization (FAO) “codex alimentarius^3^” (Supplementary Data Sheet 1). Here, we excluded images no. 11 (cheese and cold meat platter), 150, and 294 (popcorn as it could be sweet or salty) due to ambiguity. We also excluded images nos. 134 and 137 as they depicted the same chocolate muffin as in image no. 80, only from a different perspective, as well as no. 306 showing roast potatoes, which we found not clearly identifiable. Additionally, we unified the German naming of the stimuli (Supplementary Data Sheet 1) as through original description, we could not identify all images depicting similar or identical food items.

### Dietary Fiber Extension for the Food-Pics_Extended Database

Fiber content of the remaining Food-pics stimuli no. 1–568 (*n* = 562) was evaluated by four non-expert raters. These 562 stimuli were selected for further evaluation because of available macronutrient information. Dietary fiber reference nutritional information stemmed from various nutrition databases (e.g., FoodData Central App by the USDA, Swiss Food Composition Database or Food Database GmbH), online producer declarations and nutrition science books (Elmadfa et al., 2015). The respective sources of each rater and the derived fiber content per 100 g are provided (Supplementary Data Sheet 2). Mean fiber per 100 g and per item across four raters was computed. Inter-rater reliability across four raters was good overall [using psych() intraclass correlations and two-way random effects model: ICC2 = 0.76, 95% CI: 0.74, 0.79]. Yet, to ensure plausibility of the fiber ratings, we checked those ratings with larger standard deviation than the mean across the four raters (*n* = 44 items). The inter-rater variability in fiber ratings among these identified items was curated for some (*n* = 9 items due to unreliable sources) and deemed valid nonetheless for all others (*n* = 35 items) because of the following reasons: (i) visual assessment only of depicted item and its ingredients leading to an approximation of the exact ingredients (e.g., whole-grain or white flour in bakery items), (ii) imprecision of matching the depicted food item to a food database entry due to multi-component items (e.g., piece of layered caked or sushi rolls), or (iii) variation in the databases for similar or identical items (e.g., Magnum Classic).

After curation, inter-rater reliability across four raters improved slightly, but remained good overall (ICC2 = 0.77, 95% CI: 0.75, 0.80).

In order to add the fiber value to the original Food-pics_extended table, we also calculated the total fiber amount for the depicted food portion based on the provided portion size (grams_total).

**FIGURE 1:**
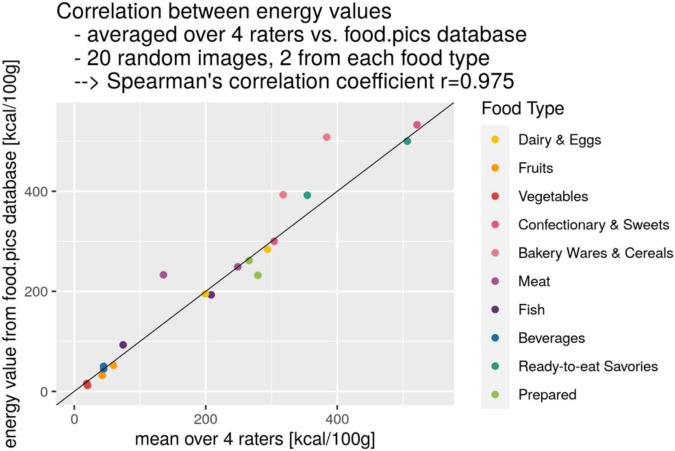
Correlation of calorie content taken as mean over four raters from different nutrient sources and from the original Food-pics database in kcal/100 g (Spearman’s rank correlation) for 20 randomly chosen food items across all 10 food types.

Compared to the nutrient information provided for the original Food-pics database, using exclusively one database,^4^ we validated reliability of extracted nutrient information for the same items from different sources. For 20 randomly chosen items across all ten food types, we found very high reliability of energy values (kcal/100 g) between the mean value of four raters from four different sources and the original energy value (Spearman’s r = 0.975, Figure 1).

## Results

**FIGURE 2:**
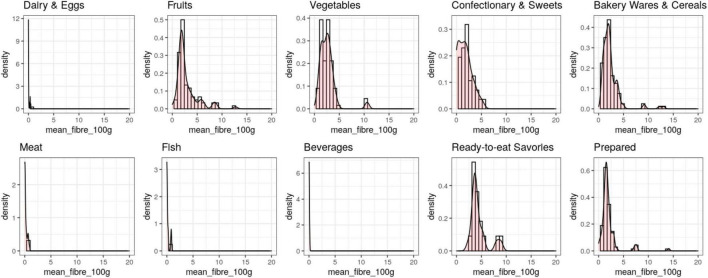
Distribution of mean fiber per 100 g by the 10 WHO food categories.

On average, fiber content was high in ready-to-eat savories (such as nuts or pretzels), fruits and vegetables, and low in dairy and eggs, fish, meat and beverages (Table 1, also note the differences in SD range and skewness, Figure 2). Note, that coefficients of variation were highest for those food categories with lower mean fiber ratings. Fiber ratings overall are not normally distributed (skewness > 9), yet log-transformation seems an adequate adjustment (skewness < 1). Fiber ratings per food category were not normally distributed, but skewed to lower fiber ratings (skewness all > 1.5 except for “Fish,” Figure 2).

**TABLE 1:** Average values, Coefficient of Variation (CoV), and skewness of fiber per 100 g of the 10 WHO food categories.

Food category/type	Fiber per 100 g (mean ± sd) (g)	CoV (σ /μ * 100) (%)	Skewness
1. Dairy and eggs	0.087 ± 0.18	206.9	1.6
2. Fruits	2.91 ± 2.24	77.0	2.2
3. Vegetables	2.651.78	67.2	2.0
4. Confectionery and sweets	1.60 ± 1.38	86.3	6.6
5. Bakery wares and cereals	2.15 ± 1.83	85.1	1.8
6. Meat	0.18 ± 0.30	166.7	3.7
7. Fish	0.13 ± 0.33	253.8	0.3
8. Beverages	0.0350.04	114.3	2.1
9. Ready-to-eat savories	4.49 ± 1.58	35.2	2.2
10. Prepared foods	1.992.03	102.0	5.6

**FIGURE 3:**
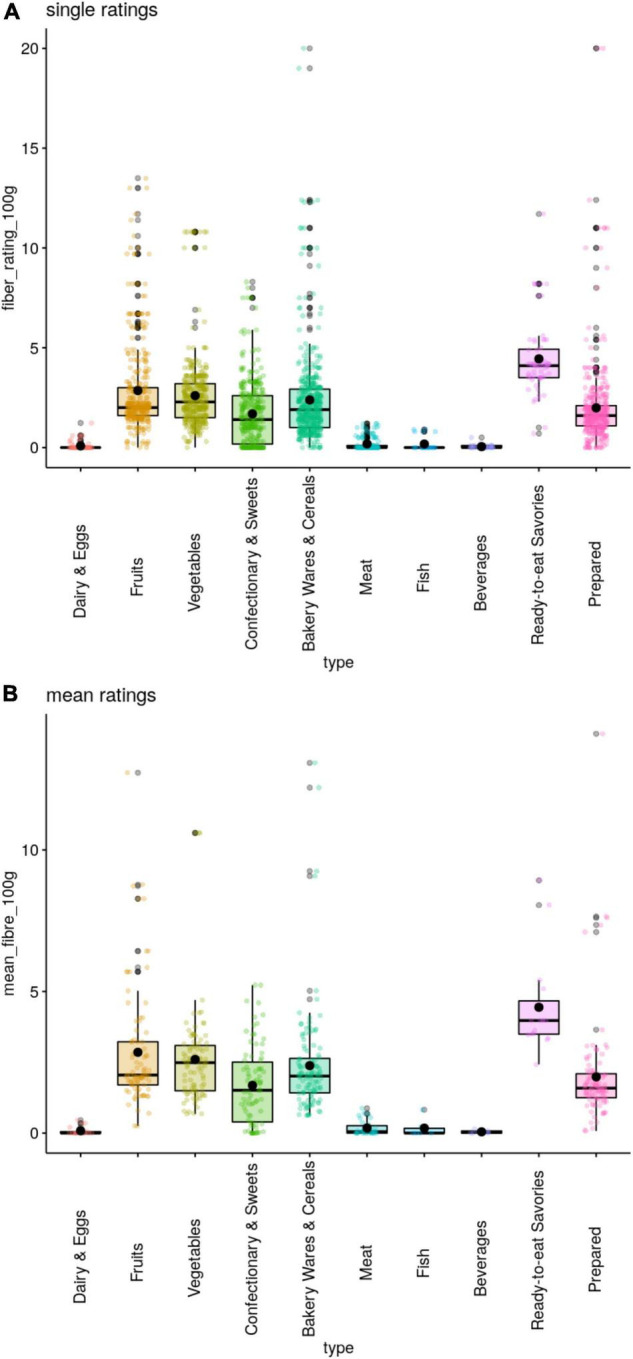
Boxplots of fiber per 100 g by the 10 WHO food categories by **(A)** single ratings per rater (top panel) and **(B)** mean ratings across all raters (bottom panel). Lines depicting median, circle depicting mean. Data falling outside the Q1–Q3 range are plotted as outliers.

Overall, single ratings by the raters and mean ratings reflect similar trends by food type (Figure 3), yet unsurprisingly outliers are less for mean ratings. Outliers in single ratings mostly occur with more than one available rating and were previously qualitatively checked and curated if needed (see section “Methods”). The remaining outliers in the single ratings are deemed accurate. For mean ratings, outliers depict high fiber ratings that are non-typical within the respective food category, e.g., artichoke (type 3 “Vegetables”: 11 g per 100 g), crisp bread with cooked ham (type 10 “Prepared”: 14.1 g per 100 g) or cheese platter with fruits (type 1 “Dairy and Eggs”: 0.5 g per 100 g).

## Discussion

We here provide referenced information on dietary fiber to the well-established Food-pics database as well as an additional categorization by WHO standards. The fiber extension reveals a relatively high fiber content of ready-to-eat savories, fruits, vegetables and bakery wares and cereals. On the other hand, food including dairy and eggs, meat and fish were not labeled high in dietary fiber due to the absence of fiber in animal-based products. Further, dietary fiber is practically not present in beverages which is also well reflected in our fiber estimations. Our averaged fiber ratings are in addition to the previously published nutrient values of the original database and may not reflect perfectly coherent nutrient estimates. We therefore call for the careful use of these ratings as proxies for fiber and not absolute nutrient levels. In the future, more detailed descriptives and more accurate nutrient ratings should be included in stimulus databases. We further identified images that were practically not usable and re-evaluated food categories. In sum, based on the robustness of our approach, we provide a valuable addition to the database “Food-pics_extended” and hope to fuel future studies in fiber-related nutrition and eating-behavior research.

## Data Availability Statement

The datasets presented in this study can be found in online repositories. The names of the repository/repositories and accession number(s) can be found in the article/Supplementary Material.

## Author Contributions

All authors listed have made a substantial, direct, and intellectual contribution to the work, and approved it for publication.

## Conflict of Interest

The authors declare that the research was conducted in the absence of any commercial or financial relationships that could be construed as a potential conflict of interest.

## Publisher’s Note

All claims expressed in this article are solely those of the authors and do not necessarily represent those of their affiliated organizations, or those of the publisher, the editors and the reviewers. Any product that may be evaluated in this article, or claim that may be made by its manufacturer, is not guaranteed or endorsed by the publisher.

## Footnotes

1
https://ec.europa.eu/food/safety/labelling-and-nutrition/food-information-consumers-legislation/nutrition-labelling_en

2
https://www.fda.gov/food/food-labeling-nutrition/questions-and-answers-dietary-fiber

3
https://www.fao.org/gsfaonline/foods/index.html

4
https://fddb.info/
